# Accurately characterizing the importance of wave‐particle interactions in radiation belt dynamics: The pitfalls of statistical wave representations

**DOI:** 10.1002/2016JA022618

**Published:** 2016-08-25

**Authors:** Kyle R. Murphy, Ian R. Mann, I. Jonathan Rae, David G. Sibeck, Clare E. J. Watt

**Affiliations:** ^1^ NASA Goddard Space Flight CentreGreenbeltMarylandUSA; ^2^ Department of PhysicsUniversity of AlbertaEdmontonAlbertaCanada; ^3^ Department of Space and Climate Physics, Mullard Space Science LaboratoryUniversity College LondonDorkingUK; ^4^ Department of MeteorologyUniversity of ReadingReadingUK

**Keywords:** ULF waves, radiation belts, geomagnetic storms, wave‐particle interactions, modeling

## Abstract

Wave‐particle interactions play a crucial role in energetic particle dynamics in the Earth's radiation belts. However, the relative importance of different wave modes in these dynamics is poorly understood. Typically, this is assessed during geomagnetic storms using statistically averaged empirical wave models as a function of geomagnetic activity in advanced radiation belt simulations. However, statistical averages poorly characterize extreme events such as geomagnetic storms in that storm‐time ultralow frequency wave power is typically larger than that derived over a solar cycle and *Kp* is a poor proxy for storm‐time wave power.

## Key Points

- Storm‐time wave power varies significantly and is typically larger than that of empirical models derived from extended data sets
- Geomagnetic activity indices are a poor proxy for storm‐time wave power
- More accurate empirical models of wave power are required

## 1 Introduction

The physical processes controlling the dynamics of the Earth's radiation belts are a fundamental and poorly understood aspect of magnetospheric physics and space weather. In general, the radiation belts exist in a quiescent state. However, during extreme conditions such as geomagnetic storms the radiation belts become exceedingly dynamic. During these storms the flux of high‐energy particles can vary by over 5 orders of magnitude during periods of either rapid electron loss or acceleration. Despite being discovered over 50 years ago [*Van Allen and Frank*, 1959] and being extensively studied [e.g., *Vampola et al*., 1992; *Baker and Blake*, 2012; *Baker et al*., 2013], the physical processes controlling the dynamics of the radiation belts and their relative contribution to electron loss and acceleration during geomagnetic storms remain an unsolved problem in magnetospheric physics.

Though poorly understood, wave‐particle interactions within the magnetosphere play a fundamental role in both electron loss and acceleration during geomagnetic storms [e.g., *Mauk et al*., 2013]. To quantify the relative contribution of electron loss and acceleration by various wave modes requires detailed studies of the electron and wave dynamics as well as global radiation belt modeling during geomagnetic storms. In general, empirical ultralow frequency (ULF) [*Brautigam and Albert*, 2000; *Ozeke et al*., 2014a; *Ali et al*., 2015] and very low frequency (VLF) wave models [*Meredith et al*., 2003; *Summers*, 2005; *Li et al*., 2009] characterized by geomagnetic indices are used as inputs for radiation belt models [*Bourdarie et al*., 2005; *Shprits and Thorne*, 2004; *Shprits et al*., 2006; *Albert et al*., 2009; *Subbotin et al*., 2011; *Reeves et al*., 2012; *Tu et al*., 2013; *Ozeke et al*., 2014a, 2014b]. The relative importance of ULF and VLF wave modes in radiation belt dynamics is then inferred from the modeled response as compared to in situ observations. This approach is valid if and only if the empirical wave models are an accurate representation of storm‐time wave amplitudes and distributions throughout the magnetosphere.

In this commentary we compare actual hourly ULF wave power during geomagnetic storms with that obtained from a typical empirical model as an illustrative example for storm‐time wave power in general. In general, storm‐time ULF wave power differs significantly from the statistically averaged ULF wave power derived over a solar cycle, varying by as much as 6 orders of magnitude. We further discuss the impacts this has on the relative importance of magnetospheric waves in radiation belt electron loss and acceleration and suggest a refined approach to quantifying the role of wave‐particle interactions in radiation belt dynamics and modeling.

## 2 ULF Wave Power

In this section we compare an empirical model of ULF wave power that has been constructed as a function of the geomagnetic *Kp* index from an entire solar cycle of data [*Ozeke et al*., 2014a] with the actual ULF wave power observed during 105 geomagnetic storms [*Murphy et al*., 2015]. The ULF wave power used in this comparison is from a database of hourly ULF wave power from the Canadian Array for Realtime Investigations of Magnetic Activity (CARISMA) [*Mann et al*., 2008] magnetometer network. *Pahud et al*. [2009] provide a detailed description of the hourly ULF wave power calculation. In this study we use dayside (6–18 magnetic local time) east‐west (D component) ULF wave power summed between frequencies of *f*_0_ = 0.83 and *f*_1_ = 15.83 mHz (∑PSD) from the Gillam magnetometer station which maps close to geosynchronous orbit and the outer edge of the outer radiation belt. Assuming the ground‐based D component ULF power spectral density (PSD) can be mapped to an azimuthal equatorial electric field PSD using a standing Alfven wave approximation [cf. *Ozeke et al*., 2009] the summed D component ULF PSD can be used as a proxy for the strength of the ULF electric field diffusion coefficient in the equatorial plane.

**Figure 1:**
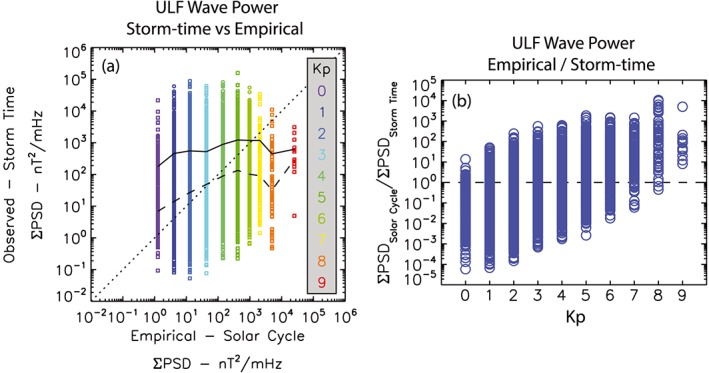
A comparison between the observed storm‐time and solar cycle averaged ∑PSD3 as a function of *Kp*. (a) Observed storm‐time versus empirical solar cycle average ∑PSD, overplotted are the median (dashed) and mean (solid) storm‐time ULF PSDs, ∑PSD, as a function of *Kp*. (b) The ratio of solar cycle ∑PSD to storm‐time ∑PSD as a function of *Kp*.

Figure 1 presents a comparison between the observed storm‐time and solar cycle averaged ∑PSD as a function of *Kp*. Figure 1a shows observed storm‐time power versus the solar cycle average, overplotted is the median (dashed) and mean (solid) storm‐time ULF PSDs during storms and the 1:1 line (dotted). In the empirical model solar cycle averaged ∑PSD is characterized by the median PSD as a function of *Kp* from *Ozeke et al*. [2012] [see also *Ozeke et al*., 2014a]. Evident in Figure 1a is that the ∑PSD varies by several orders of magnitude for all values of *Kp*. At low *Kp*, storm‐time ∑PSD is typically larger than the empirical solar cycle averages, as shown by the median and mean values lying above the dotted line. At higher *Kp* the storm‐time coefficients are comparable to the solar cycle averages. On average, as *Kp* increases so too does the ∑PSD evidenced by the positive slope of both the median (dashed) and mean (solid) lines.

Figure 1b further highlights the differences between storm‐time and solar cycle ULF wave power by comparing the absolute variations as a function of *Kp* and storm phase. Plotted in Figure 1b is the ratio of solar cycle to storm‐time ∑PSD. Figure 1b shows the same trends as Figure 1a as well as demonstrates how the absolute values vary as a function of *Kp*. During periods of low *Kp* storm‐time ULF wave power as observed by ground‐based magnetometers can at times be 4 orders of magnitude higher than the solar cycle averages; similarly at high *Kp* storm‐time ULF wave power can at times be 4 orders of magnitude lower than the solar cycle averages.

## 3 Discussion and Summary

Wave‐particle interactions play a fundamental role in radiation belt dynamics driving both radiation belt loss and acceleration. However, the relative importance of the various wave modes such as ULF, VLF, and electromagnetic ion cyclotron (EMIC) waves for driving radiation belt particle dynamics remains an unsolved problem in magnetospheric physics. Typically, this is assessed using an empirical prescription of wave power in radiation belt models. For example, ULF radial diffusion is typically characterized from empirical models based upon *Kp* [e.g., *Brautigam and Albert*, 2000; *Brautigam et al*., 2005; *Ali et al*., 2015; *Ozeke et al*., 2014a]. However, these empirical models do not characterize ULF radial diffusion for *Kp* > 7 because of low statistics, yet these are exactly the conditions which are often associated with geomagnetic storms. Further, as shown in the previous section, the fact that *Kp* is a poor indicator of ULF wave power means it is also a poor indicator of any derived ULF radial diffusion coefficient. Thus, it is difficult to assess the relative importance of the effects of ULF waves in storm‐time radiation belt dynamics definitively as compared to effects from other wave modes using models incorporating only empirical ULF diffusion coefficients. This is likely to be true of any wave mode, ULF [*Brautigam et al*., 2005; *Ali et al*., 2015; *Ozeke et al*., 2014a], VLF [*Meredith et al*., 2003; *Summers*, 2005; *Li et al*., 2009], or EMIC [*Usanova et al*., 2012], characterized empirically as a function of a geomagnetic index especially when such empirical wave power models have not specifically addressed storm‐time activity.

As illustrated in Figure 1, storm‐time ULF wave power can vary by over 6 orders of magnitude for any value of *Kp* (Figure 1a) and can be up to 4 orders of magnitude smaller or larger than the empirical ULF wave power derived over an entire solar cycle (Figure 1b). The significant spread in both panels of Figure 1 demonstrates that due to the very large variability in the observed ULF wave power *Kp* is a poor proxy for storm‐time ULF wave power. In particular, for any individual geomagnetic storm the actual diffusion rates could be 4 orders of magnitude smaller or larger than those predicted by an empirical model derived as a function of *Kp*.

In general, as illustrated by the median and mean ULF wave power in Figure 1a, storm‐time ULF wave power will be expected to be larger than that determined from both quiet‐times and storm‐times over a solar cycle's worth of data during periods of *Kp* ≤ 6 and smaller during periods of *Kp* > 6. Consequently, such models will underestimate the effects arising from ULF wave‐driven transport during moderate conditions, such as the storm recovery phase, and overestimate such effects during extremely active conditions, such as the storm sudden commencement or very large storms. This includes the magnitude of ULF wave‐driven losses, driven by outward transport and ULF wave enhanced magnetopause shadowing, as well as ULF wave‐driven acceleration due to inward ULF wave radial diffusion occurring during geomagnetic storms.

Fundamentally, Figure 1 illustrates that a more accurate description of ULF wave power and the resulting diffusion coefficients is required to (a) accurately reflect the strength of the wave‐particle interactions occurring during any particular storm and (b) assess the relative importance of ULF waves in radiation belt dynamics. Any new model of ULF wave power should be based on known drivers of ULF wave power such as enhanced solar wind dynamic pressure [*Kepko et al*., 2002], velocity [*Mann et al*., 1999], include an assessment of the potential impacts of southward interplanetary magnetic field [*Prikryl et al*., 1998], and potentially take into account the storm phase [e.g., *Murphy et al*., 2015]. Though not examined here, storm‐time variations of the dependence of wave power on solar wind and geomagnetic indices might also be seen for other wave modes such as VLF chorus and EMIC waves as well. Future studies should examine the validity of empirical representations of the power in other wave modes derived as a function of geomagnetic indices as well [e.g., *Meredith et al*., 2003; *Li et al*., 2009] including examining how such wave power might vary during storm and non‐storm‐times.

Ultimately, the most accurate representation of storm‐time wave power can be obtained using storm‐specific wave observations rather than any proxy or empirical model for wave power. Such approaches are already being implemented. For instance, *Mann et al*. [2016] used observed ULF wave power as opposed to statistical models to derive ULF wave radial diffusion coefficients, demonstrating that when using a model derived from only storm‐time observations, a simple 1‐D diffusion model accurately reproduced the enigmatic third radiation belt with no need for local acceleration via VLF waves. Similar studies [e.g., *Thorne et al*., 2013; *Tu et al*., 2014] have incorporated a proxy for event‐specific VLF wave power [*Li et al*., 2013] in order to determine the role of VLF waves in local acceleration of radiation belt electrons. Such storm‐time specific approaches are key for accurately assessing the relative importance of various loss and acceleration mechanisms during individual storms. Despite the need for and insight that storm‐time specific studies and models offer, accurate empirical models of storm‐time wave power are still needed to develop robust forecasts and nowcasts of radiation belt dynamics during geomagnetic storms. Our analysis showing that models of wave power require further refinement and better parameterizations, and most importantly, the differences between storm‐time and non‐storm‐times wave characteristics must be included if we are to understand and better predict radiation belt dynamics. Finally, any new empirical wave models or development of storm‐specific wave power should be constructed to have the same (or similar) format as existing models. In this way both event‐specific wave power and new empirical modes would be easily integrated into existing radiation belt.

## References

[jgra52838-bib-0001] Albert, J. M. , N. P. Meredith , and R. B. Horne (2009), Three‐dimensional diffusion simulation of outer radiation belt electrons during the 9 October 1990 magnetic storm, J. Geophys. Res., 114, A09214, doi:10.1029/2009JA014336.

[jgra52838-bib-0002] Ali, A. F. , S. R. Elkington , W. Tu , L. G. Ozeke , A. A. Chan , and R. H. W. Friedel (2015), Magnetic field power spectra and magnetic radial diffusion coefficients using CRRES magnetometer data, J. Geophys. Res. Space Physics, 120, 973–995, doi:10.1002/2014JA020419.

[jgra52838-bib-0003] Baker, D. N. , and J. B. Blake (2012), SAMPEX: A long‐serving radiation belt sentinel, Geophys. Monogr. Ser., 199, 21–40, doi:10.1029/2012GM001368.

[jgra52838-bib-0004] Baker, D. N. , et al. (2013), The Relativistic Electron‐Proton Telescope (REPT) instrument on board the Radiation Belt Storm Probes (RBSP) spacecraft: Characterization of Earth's radiation belt high‐energy particle populations, Space Sci. Rev., 179(1‐4), 337–381, doi:10.1007/s11214-012-9950-9.

[jgra52838-bib-0005] Bourdarie, S. , R. H. W. Friedel , J. Fennell , S. Kanekal , and T. E. Cayton (2005), Radiation belt representation of the energetic electron environment: Model and data synthesis using the Salammbo radiation belt transport code and Los Alamos geosynchronous and GPS energetic particle data, Space Weather, 3, S04S01, doi:10.1029/2004SW000065.

[jgra52838-bib-0006] Brautigam, D. H. , and J. M. Albert (2000), Radial diffusion analysis of outer radiation belt electrons during the October 9, 1990, magnetic storm, J. Geophys. Res., 105(A1), 291–309, doi:10.1029/1999JA900344.

[jgra52838-bib-0033] Brautigam, D. H. , G. P. Ginet , J. M. Albert , J. R. Wygant , D. E. Rowland , A. Ling , and J. Bass (2005), CRRES electric field power spectra and radial diffusion coefficients, J. Geophys. Res., 110, A02214, doi:10.1029/2004JA010612.

[jgra52838-bib-0007] Kepko, L. , H. E. Spence , and H. J. Singer (2002), ULF waves in the solar wind as direct drivers of magnetospheric pulsations, Geophys. Res. Lett., 29(8), 1197, doi:10.1029/2001GL014405.

[jgra52838-bib-0008] Li, W. , R. M. Thorne , V. Angelopoulos , J. Bortnik , C. M. Cully , B. Ni , O. LeContel , A. Roux , U. Auster , and W. Magnes (2009), Global distribution of whistler‐mode chorus waves observed on the THEMIS spacecraft, Geophys. Res. Lett., 36, L09104, doi:10.1029/2009GL037595.

[jgra52838-bib-0009] Li, W. , B. Ni , R. M. Thorne , J. Bortnik , J. C. Green , C. A. Kletzing , W. S. Kurth , and G. B. Hospodarsky (2013), Constructing the global distribution of chorus wave intensity using measurements of electrons by the POES satellites and waves by the Van Allen Probes, Geophys. Res. Lett., 40, 4526–4532, doi:10.1002/grl.50920.

[jgra52838-bib-0010] Mann, I. R. , A. N. Wright , K. J. Mills , and V. M. Nakariakov (1999), Excitation of magnetospheric waveguide modes by magnetosheath flows, J. Geophys. Res., 104(A1), 333–353, doi:10.1029/1998JA900026.

[jgra52838-bib-0011] Mann, I. R. , et al. (2008), The upgraded CARISMA magnetometer array in the THEMIS era, Space Sci. Rev., 141(1–4), 413–451, doi:10.1007/s11214-008-9457-6.

[jgra52838-bib-0012] Mann, I. R. , et al. (2016), Explaining the dynamics of the ultra‐relativistic third Van Allen radiation belt, Nat. Phys., doi:10.1038/nphys3799.

[jgra52838-bib-0013] Mauk, B. H. , N. J. Fox , S. G. Kanekal , R. L. Kessel , D. G. Sibeck , and A. Ukhorskiy (2013), Science objectives and rationale for the radiation belt storm probes mission, Space Sci. Rev., 179(1‐4), 3–27, doi:10.1007/s11214-012-9908-y.

[jgra52838-bib-0014] Meredith, N. P. , Horne, R. B. , Thorne, R. M. , Anderson, R. R. (2003), Favored regions for chorus‐driven electron acceleration to relativistic energies in the Earth's outer radiation belt, Geophys. Res. Lett., 30(16), 1871, doi:10.1029/2003GL017698.

[jgra52838-bib-0015] Murphy, K. R. , I. R. Mann , and D. G. Sibeck (2015), On the dependence of storm time ULF wave power on magnetopause location: Impacts for ULF wave radial diffusion, Geophys. Res. Lett., 42, 9676–9684, doi:10.1002/2015GL066592.

[jgra52838-bib-0016] Ozeke, L. G. , I. R. Mann , and I. J. Rae (2009), Mapping guided Alfvén wave magnetic field amplitudes observed on the ground to equatorial electric field amplitudes in space, J. Geophys. Res., 114, A01214, doi:10.1029/2008JA013041.

[jgra52838-bib-0017] Ozeke, L. G. , I. R. Mann , K. R. Murphy , I. J. Rae , D. K. Milling , S. R. Elkington , A. A. Chan , and H. J. Singer (2012), ULF wave derived radiation belt radial diffusion coefficients, J. Geophys. Res., 117, A04222, doi:10.1029/2011JA017463. 10.1002/2013JA019204 PMC4497482 26167440

[jgra52838-bib-0018] Ozeke, L. G. , I. R. Mann , K. R. Murphy , I. J. Rae , D. K. Milling , S. R. Elkington , A. A. Chan , and H. J. Singer (2014a), Analytic expressions for ULF wave radiation belt radial diffusion coefficient, J. Geophys. Res. Space Physics, 117, 751–760, doi:10.1029/2011JA017463. 10.1002/2013JA019204 PMC4497482 26167440

[jgra52838-bib-0019] Ozeke, L. G. , I. R. Mann , D. L. Turner , K. R. Murphy , A. W. Degeling , I. J. Rae , and D. K. Milling (2014b), Modeling cross L shell impacts of magnetopause shadowing and ULF wave radial diffusion in the Van Allen belts, Geophys. Res. Lett., 41, 6556–6562, doi:10.1002/2014GL060787.

[jgra52838-bib-0020] Pahud, D. M. , I. J. Rae , I. R. Mann , K. R. Murphy , and V. Amalraj (2009), Ground‐based Pc5 ULF wave power: Solar wind speed and MLT dependence, J. Atmos. Sol. Terr. Phys., 71(10‐11), 1082–1092, doi:10.1016/j.jastp.2008.12.004.

[jgra52838-bib-0021] Prikryl, P. , R. A. Greenwald , G. J. Sofko , J. P. Villain , C. W. S. Ziesolleck , and E. Friis‐Christensen (1998), Solar‐wind‐driven pulsed magnetic reconnection at the dayside magnetopause, Pc5 compressional oscillations, and field line resonances, J. Geophys. Res., 103(A8), 17,307–17,322, doi:10.1029/97JA03595.

[jgra52838-bib-0022] Reeves, G. D. , Y. Chen , G. S. Cunningham , R. W. H. Friedel , M. G. Henderson , V. K. Jordanova , J. Koller , S. K. Morley , M. F. Thomsen , and S. Zaharia (2012), Dynamic radiation environment assimilation model: DREAM, Space Weather, 10, S03006, doi:10.1029/2011SW000729.

[jgra52838-bib-0023] Shprits, Y. Y. , and R. M. Thorne (2004), Time dependent radial diffusion modeling of relativistic electrons with realistic loss rates, Geophys. Res. Lett., 31, L08805, doi:10.1029/2004GL019591.

[jgra52838-bib-0024] Shprits, Y. Y. , R. M. Thorne , R. Friedel , G. D. Reeves , J. Fennell , D. N. Baker , and S. G. Kanekal (2006), Outward radial diffusion driven by losses at magnetopause, J. Geophys. Res., 111, A11214, doi:10.1029/2006JA011657.

[jgra52838-bib-0025] Subbotin, D. A. , Y. Y. Shprits , and B. Ni (2011), Long‐term radiation belt simulation with the VERB 3‐D code: Comparison with CRRES observations, J. Geophys. Res., 116, A12210, doi:10.1029/2011JA017019.

[jgra52838-bib-0026] Summers, D. (2005), Quasi‐linear diffusion coefficients for field‐aligned electromagnetic waves with applications to the magnetosphere, J. Geophys. Res., 110, A08213, doi:10.1029/2005JA011159.

[jgra52838-bib-0027] Thorne, R. M. , et al. (2013), Rapid local acceleration of relativistic radiation‐belt electrons by magnetospheric chorus, Nature, 504(7480), 411–414, doi:10.1038/nature12889. 24352287 10.1038/nature12889

[jgra52838-bib-0028] Tu, W. , G. S. Cunningham , Y. Chen , M. G. Henderson , E. Camporeale , and G. D. Reeves (2013), Modeling radiation belt electron dynamics during GEM challenge intervals with the DREAM3D diffusion model, J. Geophys. Res. Space Physics, 118, 6197–6211, doi:10.1002/jgra.50560.

[jgra52838-bib-0029] Tu, W. , G. S. Cunningham , Y. Chen , S. K. Morley , G. D. Reeves , J. B. Blake , D. N. Baker , and H. Spence (2014), Event‐specific chorus wave and electron seed population models in DREAM3D using the Van Allen Probes, Geophys. Res. Lett., 41, 1359–1366, doi:10.1002/2013GL058819.

[jgra52838-bib-0030] Usanova, M. E. , I. R. Mann , J. Bortnik , L. Shao , and V. Angelopoulos (2012), THEMIS observations of electromagnetic ion cyclotron wave occurrence: Dependence on *AE*, *SYMH*, and solar wind dynamic pressure, J. Geophys. Res., 117, A10218, doi:10.1029/2012JA018049.

[jgra52838-bib-0031] Vampola, A. L. , J. V. Osborn , and B. M. Johnson (1992), CRRES magnetic electron spectrometer AFGL‐701‐5A (MEA), J. Spacecr. Rockets, 29(4), 592–595, doi:10.2514/3.25504.

[jgra52838-bib-0032] Van Allen, J. A. , and L. A. Frank (1959), Radiation around the Earth to a radial distance of 107,400 km, Nature, 183(4659), 430–434, doi:10.1038/183430a0.

